# The Evolution of Ultrasound in Medicine: A Case Report of Point-of-care Ultrasound in the Self-diagnosis of Acute Appendicitis

**DOI:** 10.5811/cpcem.2020.7.48158

**Published:** 2020-09-03

**Authors:** Barry Knapp, Kean Feyzeau, Austin Smith, Donald Byars, Craig Goodmurphy, Matt Jones

**Affiliations:** * Eastern Virginia Medical School, Department of Emergency Medicine, Norfolk, Virginia; † Eastern Virginia Medical School, Department of Anatomy and Pathology, Norfolk, Virginia

**Keywords:** ultrasound, point-of-care, appendicitis, education, medical school

## Abstract

**Introduction:**

Point-of-care ultrasound (POCUS) education during medical school develops physicians who are properly prepared for the next generation of medicine. The authors present the case of a first-year medical student who self-diagnosed appendicitis using POCUS.

**Case Report:**

A 25-year-old, first-year medical student presented to the emergency department with lower abdominal pain. What seemed like a straightforward appendicitis presentation came with a twist; the student brought self-performed ultrasound imaging of his appendix.

**Conclusion:**

The student’s ultrasound skill set reflects favorably on the rapid evolution of ultrasound teaching in medical education.

## INTRODUCTION

Point-of-care ultrasound (POCUS), the acquisition and interpretation of ultrasound images at the bedside, is an essential skill for the next generation of physicians. The integration of POCUS into undergraduate medical education (UGME) curricula is occurring at an accelerated rate. This no doubt reflects on the recognition that POCUS promotes patient safety, clinical efficiency, and elevates the level of care physicians can offer their patients. One benefit of early medical school integration is that students develop ultrasound skills in parallel with learning human anatomy – a critical element that accelerates sonographic learning. It cannot be overstated that the development of an early sonographic skill set is meant to augment the patient interview and clinical exam, not replace them. Each is an essential competency developed and ingrained early in medical school although through different educational pathways. Today’s medical students benefit from the ability to develop their sonographic skill set in parallel with learning the traditional pillars of medicine: inspection, palpation, percussion, and auscultation.

POCUS has become commonplace in many medical specialties and is evolving in others. The ability to evaluate and diagnose patients with emergent conditions using ultrasound is best illustrated in emergency medicine (EM). Diagnosing acute conditions such as appendicitis are well within the capabilities of ultrasound-trained clinicians. EM-trained physicians using POCUS to evaluate for appendicitis have been shown to have comparable operating characteristics (sensitivity, specificity, positive and negative predictive value) to comprehensive ultrasounds interpreted by radiologists.^1^ Although abdominal ultrasound for appendicitis is traditionally considered technically difficult, we present a case of a novice UGME sonographer self-diagnosing appendicitis. This is the first case report in the literature that readily demonstrates the rapid transition of medical school sonographic training in the classroom to a clinically relevant diagnostic tool.

## CASE REPORT

A 25-year-old first-year medical student arrived in the emergency department (ED) with 24 hours of lower abdominal pain. The pain was worse with cough, yard work, and eventually with walking. The clinical exam demonstrated right lower quadrant tenderness without peritoneal signs. His vital signs were normal except for mild tachycardia. Lab work, including urinalysis, was unremarkable.

Although the clinical presentation in this case was typical of appendicitis, in many institutions the next step is to obtain a computed tomography (CT) to confirm the diagnosis. The use of CT to diagnose appendicitis approaches 90% in some centers.^2^ Although CT in adults is more accurate than ultrasound in making the diagnosis of appendicitis (sensitivity 0.96 vs 0.85), ultrasound is an attractive alternative that can eliminate radiation exposure.^3^

In this case, what seemed like an otherwise straightforward appendicitis presentation came with a twist; the student brought self-performed ultrasound imaging of his appendix. Prior to his ED visit, the student went to our ultrasound teaching lab and was able to identify his inflamed appendix (without a proctor). He printed his ultrasound images and presented them to the ED attending physician. The images demonstrated an enlarged appendix (approximately 1 centimeter) with appendicolith (Image).

The emergency physician also used POCUS to reaffirm the sonographic diagnosis of appendicitis (noncompressible, focal ileus, tubular structure measuring greater than 6 millimeters in anterior-posterior diameter).^4^ Visualization of periappendiceal inflammation as well as an appendicolith, helped to confirm the diagnosis.

CPC-EM Capsule

What do we already know about this clinical entity?

*Diagnosing appendicitis using ultrasound is accurate and efficient. It is an advanced skill that can challenge even experienced sonographers*.

What makes this presentation of disease reportable?

*This is the first report in the literature of a medical student extrapolating a basic ultrasound skill set to self-diagnose appendicitis*.

What is the major learning point?

*The self-diagnosis of appendicitis by a 1st-year medical student demonstrates the rapid ultrasound skill development by future physicians*.

How might this improve emergency medicine practice?

*Point-of-care ultrasound taught by emergency physicians elevates the clinical skill set of future physicians*.

The patient was taken to the operating room without further imaging and had an uneventful appendectomy. Appendicitis was confirmed upon pathologic review of the specimen.

## DISCUSSION

It is clear that basic patient evaluation skills taught early in medical school become the framework students rely on during their clinical years and beyond. It is our goal to integrate the use of sonography as a standard part of a student’s history and physical exam. An integrated UGME ultrasound program helps students develop symbiotic clinical and sonographic skill sets. Students at our medical school Eastern Virginia Medical School (EVMS) have an ultrasound transducer in their hands as early as the orientation week of their first year.

The framework of sonographic teaching at our institution is structured around 10 core ultrasound skills that every student should graduate with. These core skills range from echocardiography to procedural guidance (Table).

Abdominal ultrasound education at our institution is focused primarily on common hepatobiliary and renal disorders. Although we do not formally train students to specifically image the appendix, the ultrasound skill set they develop during the first few months of training makes this an achievable endeavor.

POCUS is becoming the standard of care in many areas of clinical medicine. The early integration of POCUS into UGME curricula is the key to arming the next generation of physicians with the tools necessary to elevate the current standard of patient care.^5^

Although anecdotal, this report of the self-diagnosis of acute appendicitis by a medical student is evidence of a novice learner’s ability to rapidly extrapolate basic sonographic concepts into more advanced skills.

## CONCLUSION

The medical student’s ultrasound skills reflect favorably on the rapid evolution of ultrasound teaching in undergraduate medical education. As the use of ultrasound in the clinical setting expands for both diagnostic and procedural indications, it is important for medical schools to properly prepare future physicians.

## Figures and Tables

**Image:**
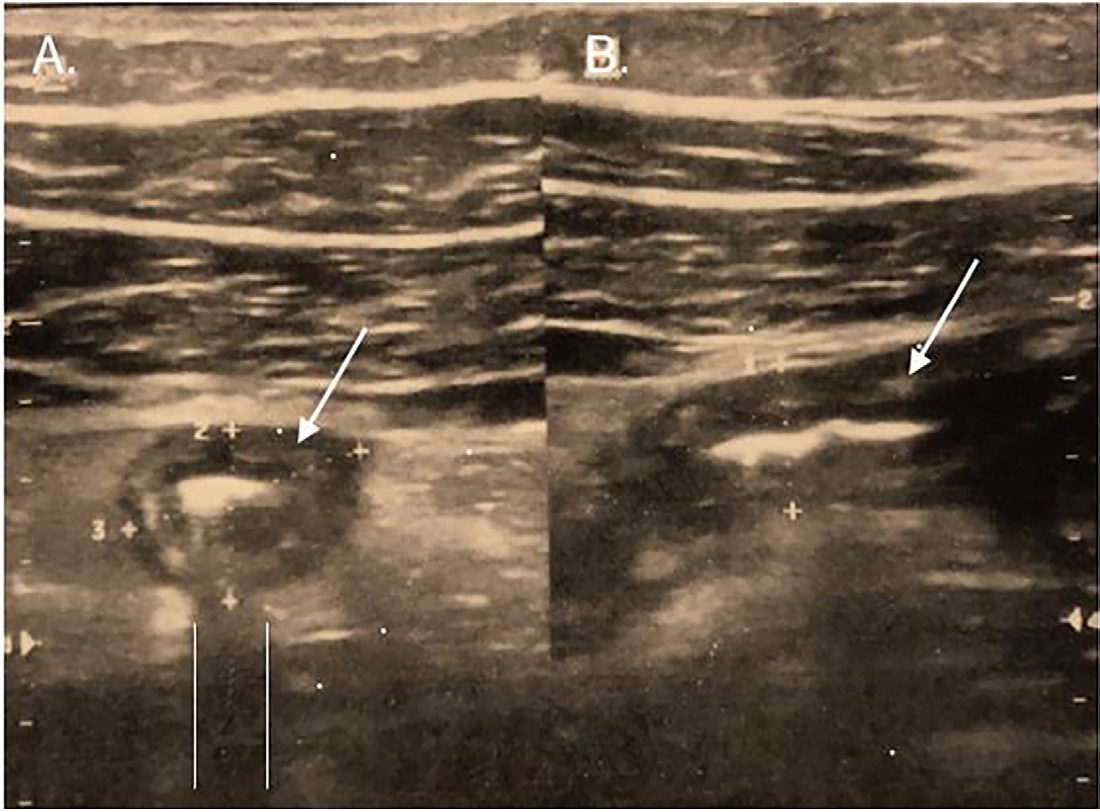
First-year medical student self-images of enlarged appendix with appendicolith in short (A) and long axis (B) are indicated by the arrows. Appendix measurements are greater than 0.9 centimeters in all dimensions. Note the bright white appendicolith causing a posterior acoustic shadow (parallel white lines) in the short axis view.

**Table:** Ten core ultrasound competencies required for medical school graduation.

Cardiac ejection fraction estimation. Cardiac evaluation for pericardial tamponade. Cardiac evaluation for right ventricular strain. Lung assessment for pneumothorax. Identification of abdominal aortic aneurysm and aortic dissection. Assessment for hemoperitoneum. Identification of cholelithiasis (gallstones). Identification of intrauterine pregnancy, gestational age and fetal lie. Assessment for common cancers (renal and liver). Establishment of ultrasound-guided vascular access.
